# Preoperative Anxiety in Chinese Adult Patients Undergoing Elective Surgeries: A Multicenter Cross-Sectional Study

**DOI:** 10.1007/s00268-022-06720-9

**Published:** 2022-09-07

**Authors:** Jiawen Yu, Yuelun Zhang, Tian Yu, Weidong Mi, Shanglong Yao, Zhen Wang, Li Xu, Yuguang Huang

**Affiliations:** 1 Department of Anesthesiology, Peking Union Medical College Hospital, Peking Union Medical College, Chinese Academy of Medical Sciences, Beijing, 100730 China; 2 Medical Research Center, Peking Union Medical College Hospital, Peking Union Medical College, Chinese Academy of Medical Sciences, Beijing, 100730 China; 3 Department of Anesthesiology, Affiliated Hospital of Zunyi Medical University, Zunyi, China; 4 Anesthesia and Operation Center, Chinese PLA General Hospital, Beijing, China; 5 Department of Anesthesiology, Union Hospital, Tongji Medical College, Huazhong University of Science and Technology, Wuhan, China; 6 Shanghai Mental Health Center, Shanghai Jiao Tong University School of Medicine, Shanghai, China

## Abstract

**Background:**

Preoperative anxiety is associated with increased use of anesthetics and poorer postoperative outcomes. However, the prevalence of preoperative anxiety has not been characterized in Chinese patients. In this study, we aimed to estimate the overall prevalence of preoperative anxiety in Chinese adult patients and to explore the sociodemographic and clinical factors associated with preoperative anxiety in China.

**Methods:**

This study was a multicenter cross-sectional study conducted at 32 tertiary referral centers in China from September 1 to October 31, 2020. Adult patients scheduled for elective surgery were evaluated by the 7-item Perioperative Anxiety Scale (PAS-7) for preoperative anxiety after entrance to the operating zone.

**Results:**

A total of 5191 patients were recruited, and 5018 of them were analyzed. The prevalence of preoperative anxiety measured by PAS-7 was 15.8% (95% CI 14.8 to 16.9%). Multivariable analyses showed female sex, younger age, non-retired, first in a lifetime surgery, surgery of higher risk, and poorer preoperative sleep were associated with higher prevalence of preoperative anxiety.

**Conclusions:**

Preoperative anxiety was relatively common (prevalence of 15.8%) among adult Chinese patients undergoing elective surgeries. Further studies are needed using suitable assessment tools to better characterize preoperative anxiety, and additional focus should be placed on perioperative education and intervention, especially in primary hospitals.

**Trial registration:**

This study was registered prospectively at www.chictr.org.cn (ChiCTR1900027639) on November 22, 2019.

## Introduction

Anxiety is common among perioperative patients [1–4]. Anxiety involves both in negative psychological expectations and physical changes toward future events [5]. In addition to contributing to an unpleasant perioperative experience, preoperative anxiety has been associated with increased use of anesthetics [6], severer postoperative pain [7], cognitive impairment [8], higher risk of postoperative complications and deaths [9], and poorer long-term quality of life and survival [10].

Approximately 40 million patients undergo surgical procedures annually in China [11], but the prevalence of preoperative anxiety has not been fully investigated. A few small-scale studies involving several hundred patients reported prevalence of preoperative anxiety ranging from 11 to 89% [8, 12–14]. However, these studies were mainly single-center studies involving only specific types of surgeries using various anxiety assessment tools. The large degree of heterogeneity among studies has precluded generalization of the results to other patient population. Therefore, a nationwide cross-sectional investigation is needed to draw a clearer picture of the existing circumstances of perioperative anxiety in China.

Multiple anxiety assessment tools have been used in clinical and research settings. However, there is no consensus assessment tool for surgical patients, and assessments are difficult to perform perioperatively. Cultural and language differences also limit the use of certain assessment tools in different countries [3, 15]. Chinese patients often do not express negative feelings directly, but rather convey their disturbed mood in the form of somatic symptoms. Therefore, somatic anxiety is an important aspect in anxiety assessments in the Chinese population.

In this study, we aimed to estimate the prevalence of preoperative anxiety in China and determine sociodemographic and clinical factors associated with preoperative anxiety.

## Methods

The study protocol was approved by Research Ethics Committees at Peking Union Medical College Hospital and registered prospectively at www.chictr.org.cn (ChiCTR1900027639). Written informed consent was obtained before data collection.

### Patients

This national multicenter cross-sectional study was conducted at 32 tertiary referral centers in China from September 1 to October 31, 2020. Recruitment of patients was completed using a cluster sampling strategy. Eligible centers were selected from membership institutes of Chinese Society of Anesthesiology (CSA) by the research expert committee based on geographic location, annual surgical volume, and representativeness of the general surgical population during several expert panel discussions. Patients who met all the following inclusion criteria were considered for recruitment: (1) patients undergoing elective surgery; (2) ASA classification I-III; (3) age ≥18 years; (4) capable of understanding and completing the questionnaire with assistance from medical staff; (5) informed consent obtained. The exclusion criteria were as follows: (1) current mental illness, disturbance of consciousness, or unable to communicate; 2) substance use disorders; (3) long-term (more than 3 months) usage of antidepressants, antianxiety medications, or other psychotropic medications; (4) could not understand, or refused to finish the questionnaire; and (5) unable to give consent, or refused to participate. Eligible patients were consecutively recruited in each center during the 2-month study period.

### Questionnaire

The 7-item Perioperative Anxiety Scale (PAS-7) was used as the major assessment tool for preoperative anxiety status. The PAS-7 was specifically designed for adult Chinese surgical patients and showed good reliability and validity compared to Generalized Anxiety Disorder-7 Scale (GAD-7) [16]. The PAS-7 contains seven questions of a 5-point Likert scale (Appendix 1), with Questions No.1-4 regarding mental anxiety and Questions No.5-7 regarding somatic anxiety [16]. Patients with a PAS-7 score ≥8 were considered to have preoperative anxiety.

### Data collection

At least two resident anesthesiologists in each participating center were trained to evaluate and collect patient information in a standardized manner. Patients who met the inclusion criteria were visited one day before surgery, during which informed consent was obtained and general patient information was collected. Sociodemographic information and medical conditions were collected via hospital information system and face-to-face inquiry. Surgical risk was assessed by the attending anesthesiologist according to the China Hospital Association (CHA) surgical risk evaluation form (Appendix 1) adapted from national nosocomial infections surveillance system (NNIS) risk index [17, 18]. On the day of surgery, the anxiety status of each patient was evaluated after separation from their companion. The patients were encouraged to complete the PAS-7 questionnaire in a private setting. Patients could seek help with any issues associated with the questionnaire, and standardized instructions and responses were used for assistance. An electronic data capture system was used for data collection and questionnaire distribution. After completion of survey, the database was locked and specific information was exported by an exclusive information extractor. Specially trained data inspectors oversaw the data collection process.

### Statistical analysis

The raw preoperative anxiety prevalence was calculated using the number of patients diagnosed with anxiety divided by the overall number of patients. A confidence interval (CI) for prevalence of anxiety was calculated using the Agresti–Coull method [19]. Raw prevalence was also calculated in subgroups according to sociodemographic and clinical characteristics. The association between preoperative anxiety and patient characteristics was analyzed using multivariable logistic regression analysis with stepwise variable selection using Akaike information criterion (AIC). Mixed-effect model analysis considering potential cluster effect from each participating center was also performed (Appendix 1). Strength of association was expressed using odds ratio (OR) with 95% CI. All data were analyzed using R (version 4.0.3, R Foundation for Statistical Computing, Vienna, Austria, 2020, https://www.R-project.org), and two-sided P<0.05 was considered as statistically significant.

Sample size was determined using methods for estimating the CI of a binomial proportion. Previous reported preoperative anxiety prevalence varied across a wide range (11 to 89%) [8, 9, 12, 13, 20, 21]. We used 50% as the estimated prevalence because the sample size was largest under this estimated value. A simple random sampling method required 402 patients with 10% absolute width of lower to upper bounds of the 95%CI. Considering the cluster sampling design, we assumed a within-cluster rate of homogeneity of 0.05. The design effect was 8.45 when the number of eligible patients was 150 in each center. Thirty-two centers would have contributed a total of 4,800 patients, which was larger than 3396 (sample size determined for simple random sampling × design effect), and satisfied the requirement for the confidence accuracy. Considering possible data missing, this study planned to include 5100 patients.

## Results

**Fig. 1:**
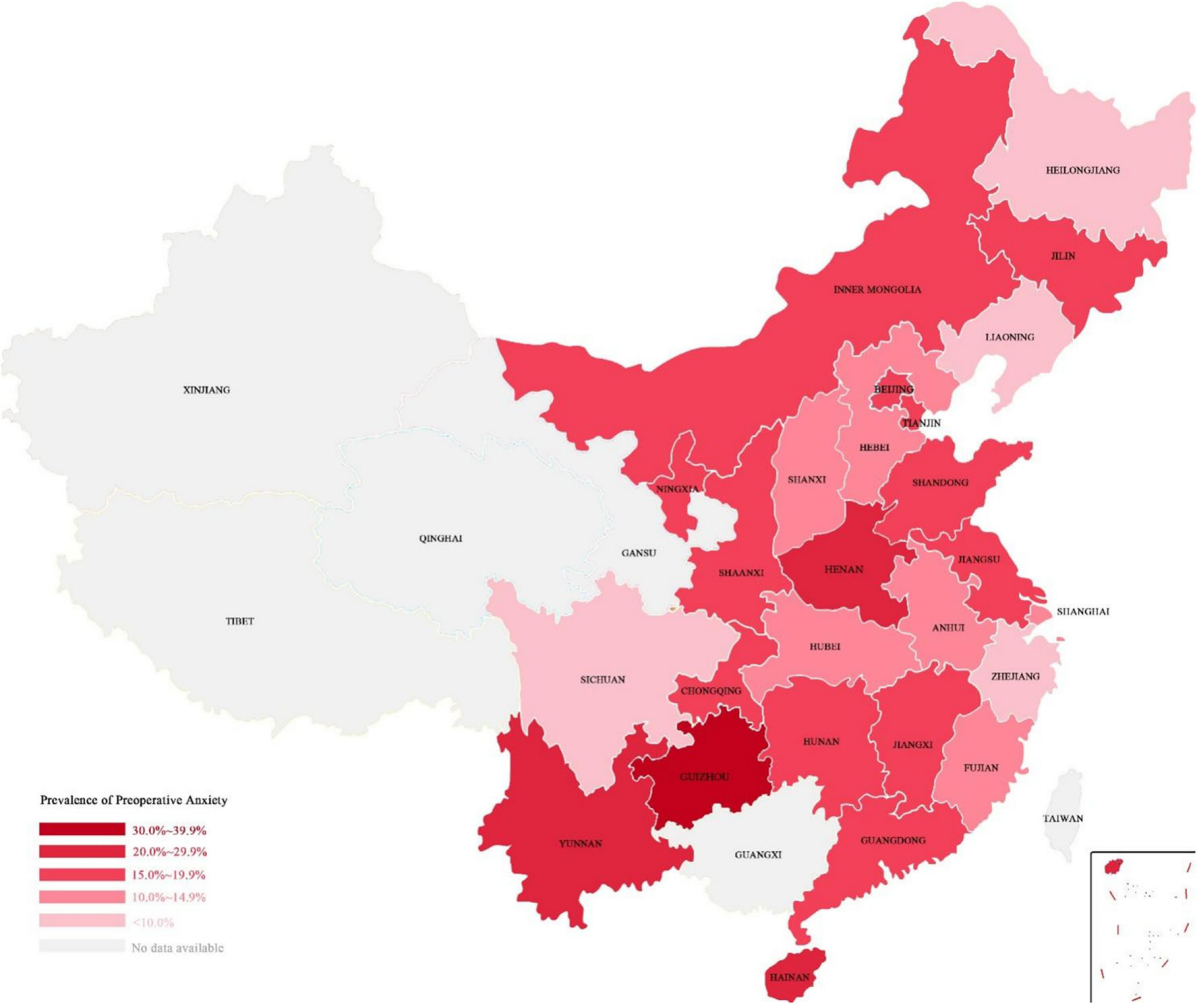
Prevalence of preoperative anxiety in China

A total of 32 tertiary hospitals in 26 provinces participated in this national cross-sectional study, including hospitals in Anhui, Beijing, Chongqing, Fujian, Guangdong, Guizhou, Hainan, Hebei, Heilongjiang, Henan, Hubei, Hunan, Inner Mongolia, Jiangsu, Jiangxi, Jilin, Liaoning, Ningxia, Shanxi, Shandong, Shanghai, Shaanxi, Sichuan, Tianjin, Yunnan, and Zhejiang Province (Fig. 1 and Appendix 2).

In total, 5,191 patients were recruited and 5,018 were included after data cleaning. Fifty-three patients were excluded due to missing information on anxiety assessment, 73 were excluded due to inappropriate timing of preoperative assessment, 24 were excluded due to missing PAS-7 score, and 23 patients were excluded for inappropriate surgical department. The recruitment rate was 96.7%. The mean age was 47.6 ± 14.4 years, and 59.0% were female. The majority of patients underwent surgery in the departments of general surgery (35.8%), gynecology (17.9%), and orthopedics (14.5%). Most surgeries were low risk (72.8%) and medium risk (22.4%), with only 4.8% considered high or very high risk. Patient characteristics are summarized in Table 1.

**Table 1:** Patients characteristics

Item	*N* (%)	Item	*N* (%)
Age (yrs.)	47.6 ± 14.4 (mean ± SD)	Quality of sleep before surgery	
[18,36]	1274 (25.5%)	Good	2131 (42.5%)
[37,48]	1251 (25.1%)	Fair	2154 (43.0%)
[49,58]	1271 (25.5%)	Poor	726 (14.5%)
[59,91]	1194 (23.9%)	History of previous surgeries	
*Sex*		No	2435 (48.6%)
Male	2055 (41.0%)	Yes	2579 (51.4%)
Female	2960 (59.0%)	Nature of the disease	
ASA* classification		Benign	2611 (52.1%)
ASA I	1448 (28.9%)	Malignant	839 (16.7%)
ASA II	3266 (65.2%)	Unknown	1563 (31.2%)
ASA III	296 (5.9%)	Anesthesia Plan	
*Marital status*		General anesthesia	4533 (90.6%)
Married	4398 (87.7%)	Neuraxial anesthesia	351 (7.0%)
Unmarried	436 (8.7%)	Nerve block	56 (1.1%)
Divorced	90 (1.8%)	Monitored anesthesia care	22 (0.4%)
Widowed	93 (1.8%)	Local anesthesia	43 (0.9%)
*Education level*		Estimated operation time	
Below high school	2084 (41.6%)	< 3 h	4159 (83.0%)
High school	1320 (26.3%)	3–9 h	846 (16.9%)
Undergraduate	1475 (29.5%)	> 9 h	7 (0.1%)
Graduate and above	132 (2.6%)	Cha*** surgical risk stratification	
*Occupational status*		0	3641 (72.8%)
Employee	1950 (38.9%)	1	1118 (22.4%)
Individual business	735 (14.6%)	2	218 (4.3%)
Retiree	1007 (20.1%)	3	24 (0.5%)
Others^**^	1324 (26.4%)	Operation sequence	
*Family monthly income*		First of the day	1155 (23.1%)
< 3000RMB	459 (9.2%)	Others	3855 (76.9%)
3000-5000RMB	934 (18.6%)	Beginning time of surgery	
5000-10000RMB	1134 (22.6%)	Morning	2844 (56.8%)
> 10000RMB	1041 (20.8%)	Afternoon	2090 (41.7%)
Unwilling to inform	1446 (28.8%)	Evening	72 (1.4%)
*Payment*		Night	5 (0.1%)
National Health Care (urban)	3380 (67.4%)	Assessment method	
National Health Care (rural)	954 (19.0%)	Patient self-assessment	3696 (73.7%)
Self-pay	571 (11.4%)	By anesthesiologist	1320 (26.3%)
Others	109 (2.2%)		

*ASA: American Society of Anesthesiologists **Including farmers, freelancers, and unemployed people. ***CHA: China Hospital Association

A total of 794 patients suffered from preoperative anxiety, as determined using the PAS-7 (PAS-7 score ≥ 8), revealing a prevalence of 15.8% (95% CI 14.8% to 16.9%) in adult Chinese surgical patients. The mean PAS-7 score was 4.03 ± 4.19 (median [IQR] 3.0 [1.0, 6.0]), with 3.20 ± 3.04 (median [IQR] 2.5 [1.0, 5.0]) for mental anxiety score and 0.83 ± 1.68 (median [IQR] 0.0 [0.0, 1.0]) for somatic anxiety score.

**Fig. 2:**
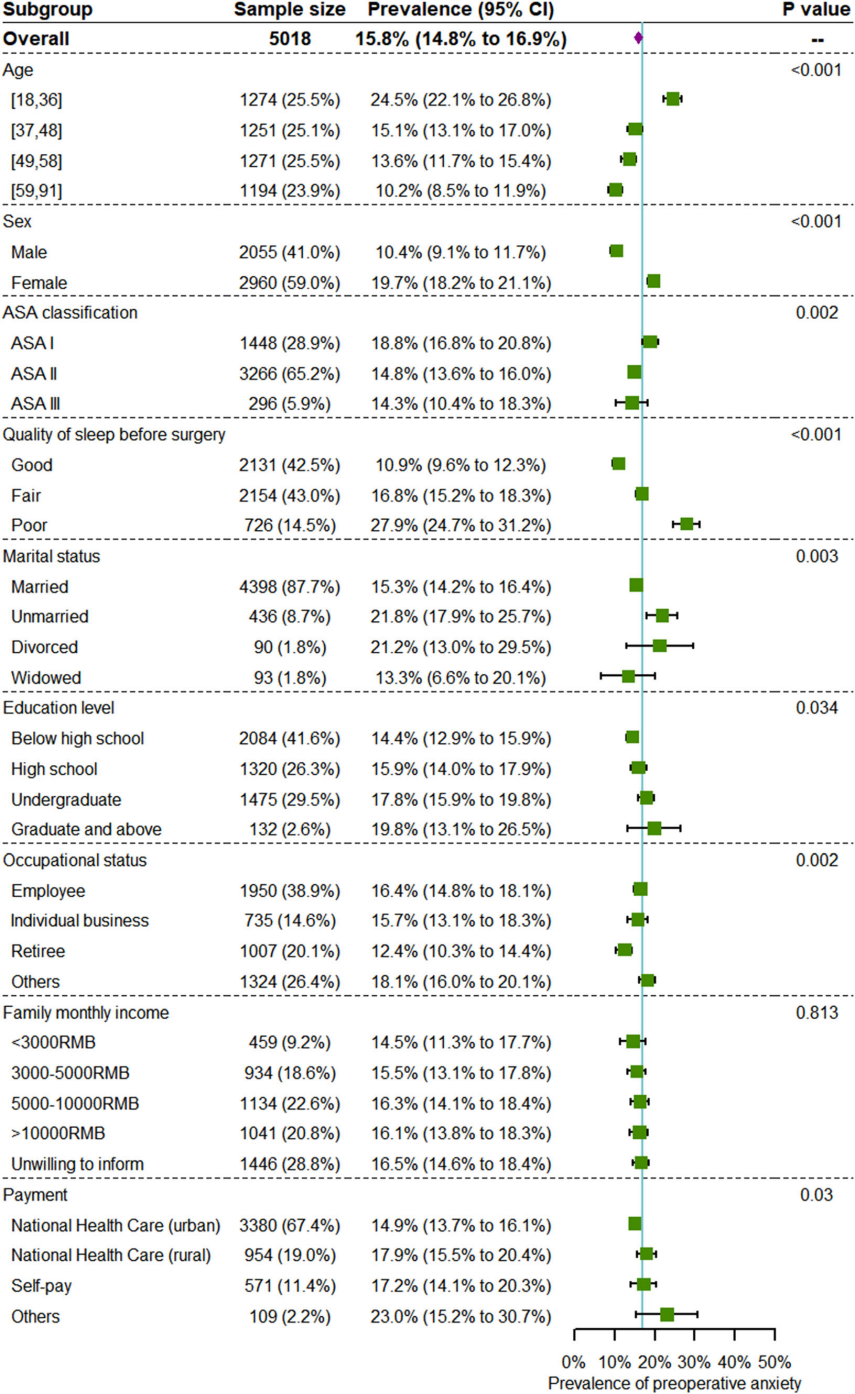
Subgroup analysis of patients’ preoperative anxiety (sociodemographic information)

Subgroup analysis was performed based on baseline and sociodemographic characteristics (Fig. 2). This analysis showed that the prevalence of preoperative anxiety was lower in senior patients. The preoperative anxiety prevalence in the youngest group was 24.5% (95% CI 22.1% to 26.8%), and the prevalence was 10.2% (95% CI 8.5% to 11.9%) in the oldest. On average, 19.7% of female patients had preoperative anxiety while only 10.4% of male patients had preoperative anxiety. Patients with lower ASA classification were more likely to suffer from preoperative anxiety. Patients who slept well before surgery had a lower prevalence of anxiety (10.9%; 95% CI 9.6% to 12.3%) than those who slept poorly (27.9%; 95% CI 24.7% to 31.2%). Unmarried and divorced patients had a higher prevalence of preoperative anxiety. Patients with higher education experience tended to be more anxious. Retired patients were less likely to present with preoperative anxiety.

**Fig. 3:**
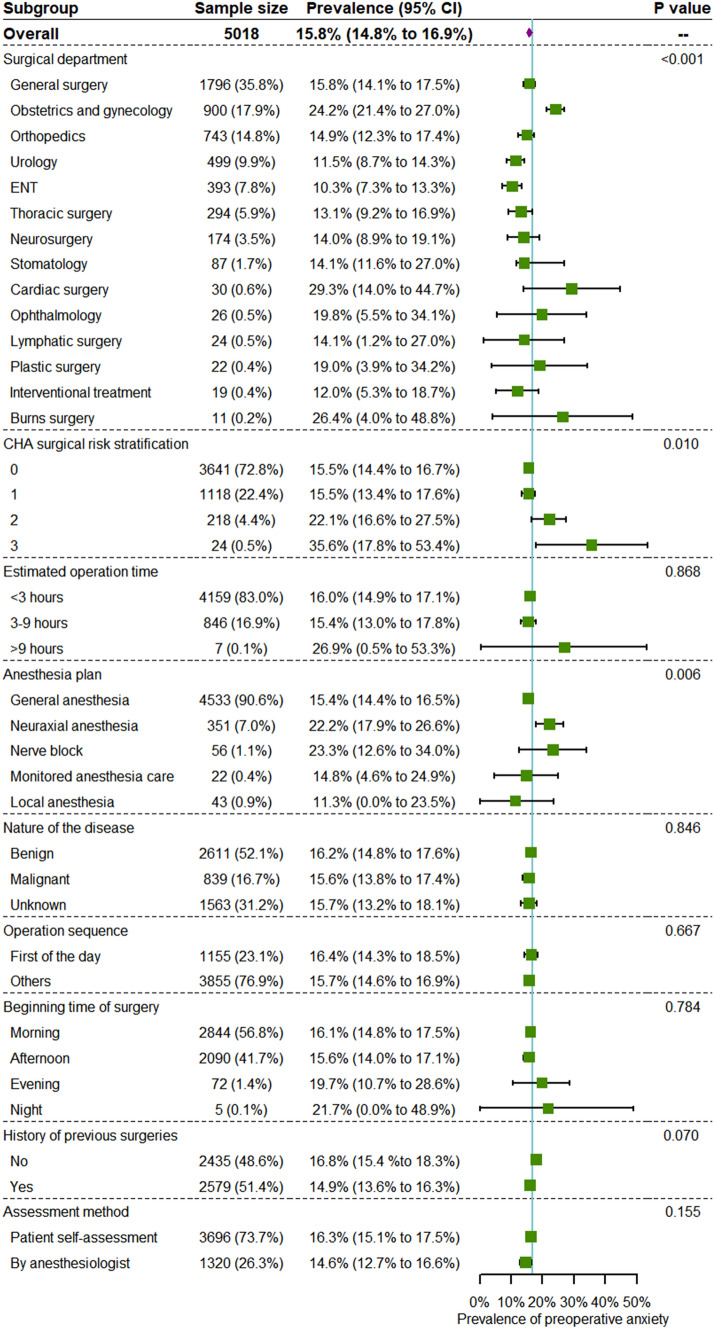
Subgroup analysis of patients’ preoperative anxiety (surgical information) *ENT: ear, nose, and throat department; CHA: China Hospital Association

Subgroup analysis according to surgical factors was also performed (Fig. 3). The prevalence of preoperative anxiety was highest (approximately 25%) among patients undergoing cardiac surgery or obstetrics and gynecology surgery. The prevalence was lowest (10.3%; 95% CI 7.3% to 13.3%) in patients undergoing ENT surgeries. Higher risk surgical procedures were associated with higher prevalence of preoperative anxiety. In terms of anesthesia plan, 15.4% (95% CI 14.4% to 16.5%) of patients undergoing general anesthesia had preoperative anxiety. Patients undergoing neuraxial anesthesia or nerve block were more likely to experience preoperative anxiety, with an average prevalence greater than 20%. Patients undergoing local anesthesia had the lowest rate of preoperative anxiety (11.3%; 95% CI 0.0% to 23.5%). Patients without history of surgery had a slightly higher prevalence of preoperative anxiety (16.8%; 95% CI 15.4% to 18.3%) than those who had previously had surgery (14.9%; 95% CI 13.6% to 16.3%). Estimated operation time, operation sequence, and nature of disease did not significantly influence preoperative anxiety.

Multivariable logistic regression showed that younger age, female sex, non-retired people, poor sleep quality, no previous history of surgery, and high-risk surgery (CHA ≥ 2) were associated with increased risk for preoperative anxiety (Table 2).

**Table 2:** Potential patients' characteristics associated with preoperative anxiety in multivariable logistic regression

Factor	OR (95% CI)	*P* value
Age (per 10 years)	0.704 (0.655 to 0.756)	< 0.001
Female	1.845 (1.546 to 2.208)	< 0.001
*Occupational status*		
Retiree	As reference	
Employee	1.516 (1.137 to 2.018)	0.004
Individual business	1.368 (0.994 to 1.883)	0.055
Others	1.292 (0.977 to 1.709)	0.072
*CHA surgical risk classification*		
0	As reference	
1	1.175 (0.965 to 1.427)	0.105
2	1.774 (1.229 to 2.518)	0.002
	2.489 (0.954 to 6.008)	0.049
*Quality of sleep before surgery*		
Good	As reference	
Normal	1.612 (1.344 to 1.937)	< 0.001
Bad	3.007 (2.413 to 3.746)	< 0.001
*Previous history of surgery*		
Yes	As reference	
No	1.217 (1.036 to 1.427)	0.016

## Discussion

This study was the largest nationwide cross-sectional investigation of preoperative anxiety in China including more than 5,000 surgical patients. The results showed that the overall prevalence of preoperative anxiety in Chinese adult patients undergoing elective surgery was 15.8%. Compared with previously reported rates of 11 to 89% [3, 14, 21, 22], the prevalence of preoperative anxiety in this study was lower than those from previous domestic [21] and overseas studies.

Several factors may have contributed to the differences between this study and others. Our study aimed to determine the overall prevalence of preoperative anxiety in all surgical patients in China. Therefore, patients undergoing diverse surgical procedures were recruited. Previous studies reported a preoperative anxiety prevalence of approximately 30% in gynecology, orthopedic, or cardiac patients [8, 10, 23–25], approximately 20% in gastrointestinal patients [13], and approximately 10% in esthetic plastic surgery patients [14]. Although nearly one-fifth of our patients were from obstetrics and gynecology departments, where prevalence of preoperative anxiety was anticipated to be high, many patients were from other departments including general surgery, orthopedics, ENT, and urology, where patients were less likely to experience preoperative anxiety. Second, this study was a cross-sectional study conducted in a 2-month period without selection of specific surgical type or patient characteristics, and reflected the actual profile of surgeries conducted in China. Therefore, the majority were low-risk surgeries, which might have contributed to the lower prevalence of preoperative anxiety. Third, this study was conducted in tertiary referral centers and teaching hospitals. Highly regarded reputations of these hospitals might have mitigated preoperative anxiety in many patients. Moreover, preoperative education by surgical team, and increased access to surgical and anesthetic knowledge on the internet may have allowed patients in this study to better understand what they might experience during the perioperative phase.

The lower prevalence of preoperative anxiety in this study indicated that previous studies focusing on certain surgical types at single centers might have overestimated preoperative anxiety. Despite the relatively low prevalence of preoperative anxiety in this cross-sectional survey, the large number of surgical patients in China suggests that millions of patients might experience preoperative anxiety annually.

Compatibility of the anxiety assessment tools used in this study with the Chinese cultural context was crucial for preoperative anxiety evaluation. The PAS-7 is a self-report scale specifically designed for Chinese surgical patients. As Chinese patients are often reserved with regard to negative psychological feelings, but more likely to express anxiety-related physical discomfort, the PAS-7 innovatively introduced three questions about physical symptoms, allowing for inclusion of somatic anxiety in the assessment.

Potential characteristics associated with preoperative anxiety included female sex, non-retired, no previous surgery experience, and high-risk surgery, which were consistent with previous studies [26]. Male patients might feel more difficult to express their anxiety due to social and cultural pressure, which could influence the results. A previous study reported preoperative anxiety prevalence up to 60% in patients with thyroid nodules waiting for pathological diagnosis [12], but no significant differences were observed in this study among patients undergoing surgery for benign lesions, malignancies, or lesions with unknown properties. In this study, we found that younger patients were more anxious preoperatively. Many studies have shown that levels of anxiety differ with age [27]. A previous systematic review suggested that patients above 55 years old were less likely to suffer from generalized anxiety disorder [28]. Younger patients, particularly those at working age, were more vulnerable to higher levels of anxiety [29]. While younger adults are often afraid of intraoperative awareness, unsuccessful anesthesia, postoperative pain, and postoperative complications which might impact their long-term quality of life, or even shorten their lifespan [2, 27], elderly patients are less likely to express their fears regarding anesthesia and surgery.

Poor quality of sleep was associated with preoperative anxiety and may be a target to reduce preoperative anxiety. Benzodiazepines and melatonin as premedication for surgical patients have been shown in the clinical setting to reduce preoperative and postoperative anxiety, resulting in improved perioperative experience and postoperative recovery [30]. However, whether poor sleep caused preoperative anxiety requires further investigation.

This study was subject to several limitations. First, although this was largest nationwide cross-sectional study of preoperative anxiety, the participating medical centers were mainly tertiary referral hospitals that provide high-quality medical care and may not reflect the conditions in secondary or primary care facilities. We also did not include hospitals in several provinces, especially in western China, where medical resources might be scarce. Future studies should focus more on primary care facilities. Second, the cluster sampling used in this study may lead to selection bias compared to multistage probability sampling. To minimize potential bias, eligible research centers were selected during several expert panel discussions, and factors such as geographic distribution, surgical volume, and surgical specialties were considered. The relatively large sample size of this study might have mitigated representative sampling concerns. Third, since the preoperative anxiety evaluation was primarily completed in waiting areas just before anesthesia, more detailed information about potential reasons for anxiety was not evaluated. Postoperative prognosis was not collected for this study, which prevented evaluation of the association between preoperative anxiety and prognosis. Fourth, the cross-sectional nature of this study precluded determination of causality. For example, we could not ascertain whether poor quality of sleep caused anxiety or anxiety caused poor sleep. Finally, patients with mental illnesses, long-term usage of psychotropic medications, or substance use disorders were excluded to minimize outliers, as the majority of surgical patients in China have no history of mental illness and condition of substance dependence is very rare. However, individuals with mental illness or substance dependence may require special psychological assessment and intervention preoperatively. Further cohort studies should address these limitations.

In conclusion, preoperative anxiety is relatively common among adult Chinese patients undergoing elective surgeries. More attention should be paid to screening and prevention of preoperative anxiety using scenario-suitable assessment tools, and perioperative education and intervention. Further clinical studies are needed to determine appropriate approaches to preoperative anxiety assessment in China, particularly in primary hospitals.

## Appendix 1

See Tables

**Table 3:** The 7-item Perioperative Anxiety Scale (PAS-7)

Item	Not at all	Slightly	Moderately	Very	Extremely
I am worried about the effect of the operation	0	1	2	3	4
I am worried about accidents during the operation	0	1	2	3	4
I am worried about the pain caused by the operation	0	1	2	3	4
Thinking about the operation makes me more nervous and worried than usual	0	1	2	3	4
Thinking about the operation makes my hands tremble	0	1	2	3	4
Thinking about the operation makes my face become hot and blushed, and my hands and feet sweat	0	1	2	3	4
Thinking about the surgery makes my breathing difficult	0	1	2	3	4

^3^,

**Table 4:** China Hospital Association (CHA) surgical risk evaluation form Item

Surgical wound classification	Score	ASA classification	Score	Surgical duration (estimated surgical duration for preoperative assessment)	Score
Clean	0	I	0	T1: within 3 h	0
Clean-contaminated	0	II	0	T2: more than 3 h	1
Contaminated	1	III	1	Surgery type	/
Dirty-infected	1	IV	1	Superficial tissue surgery	/
		V	1	Deep tissue surgery	/
		VI	1	Organ surgery	/
				Lacuna surgery	/

CHA score = Surgical wound classification score + ASA classification score + surgical duration score (Total: 0 to 3 points)

CHA score ≥2 is considered as high risk

4,

**Table 5:** Mixed-effects model considering potential cluster effect from the same hospital

Factor	OR (95%CI)	*P*
Age (per 10 years)	0.726 (0.675,0.781)	< 0.001
Female	1.825 (1.523,2.185)	< 0.001
*Occupational status*		
Retiree	As reference	
Employee	1.508 (1.129, 2.015)	0.005
Individual business	1.417 (1.024, 1.961)	0.036
Others	1.344 (1.010, 1.788)	0.043
*CHA surgical risk classification*		
0	As reference	
1	1.159 (0.945, 1.420)	0.156
2	1.823 (1.260, 2.638)	0.001
3	2.599 (1.022, 6.611)	0.045
*Quality of sleep before surgery*		
Good	As reference	
Normal	1.610 (1.336, 1.939)	< 0.001
Bad	2.940 (2.349, 3.679)	< 0.001
*Previous history of surgery*		
Yes	As reference	
No	1.239 (1.053, 1.458)	0.010

CHA: China Hospital Association

5

## List of participating hospitals

1. Affiliated Hospital of Inner Mongolia Medical College (Inner Mongolia)
2. Affiliated Hospital of Zunyi Medical College (Guizhou)
3. Anhui Provincial Hospital (Anhui)
4. Beijing Shijitan Hospital, Capital Medical University (Beijing)
5. Changhai Hospital affiliated to Naval Military Medical University (Shanghai)
6. Chongqing Southwest Hospital (Chongqing)
7. First Affiliated Hospital of Nanchang University (Jiangxi)
8. First Hospital of Jilin University (Jilin)
9. Fudan University Cancer Hospital (Shanghai)
10. Hainan Provincial People's Hospital (Hainan)
11. Hebei Provincial People's Hospital (Hebei)
12. Ningxia Medical University General Hospital (Ningxia)
13. Peking Union Medical College Hospital (Beijing)
14. Chinese PLA General Hospital (Beijing)
15. Qianfoshan Hospital of Shandong Province (Shandong)
16. Renji Hospital affiliated to Shanghai Jiao Tong University School of Medicine (Shanghai)
17. Ruijin Hospital, Shanghai Jiao Tong University School of Medicine (Shanghai)
18. Second Affiliated Hospital of Harbin Medical University (Heilongjiang)
19. The First Affiliated Hospital of Air Force Military Medical University (Shaanxi)
20. The First Affiliated Hospital of China Medical University (Liaoning)
21. The First Affiliated Hospital of Soochow University (Jiangsu)
22. The First Affiliated Hospital of Sun Yat-sen University (Guangdong)
23. The First Affiliated Hospital of Zhengzhou University (Henan)
24. The Second Affiliated Hospital of Kunming Medical College (Yunnan)
25. The Second Affiliated Hospital of Zhejiang University School of Medicine (Zhejiang)
26. The Second Hospital of Shanxi Medical University (Shanxi)
27. The Second Xiangya Hospital of Central South University (Hunan)
28. Tianjin Medical University General Hospital (Tianjin)
29. Union Hospital affiliated to Fujian Medical University (Fujian)
30. Union Hospital, Tongji Medical College, Huazhong University of Science and Technology (Hubei)
31. West China Hospital, Sichuan University (Sichuan)
32. Xuanwu Hospital, Capital Medical University (Beijing)
